# Open‐Source Simulator of Imaging Near Metal at Arbitrary Magnetic Field Strengths

**DOI:** 10.1002/mrm.70163

**Published:** 2025-11-03

**Authors:** Kübra Keskin, Ana R. Sanson, Brian A. Hargreaves, Krishna S. Nayak

**Affiliations:** ^1^ Ming Hsieh Department of Electrical and Computer Engineering University of Southern California Los Angeles California USA; ^2^ Electrical Engineering Stanford University Stanford California USA; ^3^ Radiology Stanford University Stanford California USA; ^4^ Bioengineering Stanford University Stanford California USA; ^5^ Alfred E. Mann Department of Biomedical Engineering University of Southern California Los Angeles California USA

**Keywords:** imaging near metallic implants, low‐field, metal artifact, MRI simulation, multi‐spectral imaging, open source, SEMAC, VAT

## Abstract

**Purpose:**

The increasing prevalence of orthopedic metallic implants necessitates optimization of MRI methods to monitor surrounding tissues and identify complications. The emergence of contemporary mid‐ and low‐field systems as promising platforms for imaging around metal requires development of new protocols optimized for these field strengths. Motivated by these, we develop a simulation framework that predicts MRI performance near metallic implants, considering B0 field strength and multi‐spectral imaging (MSI) parameters.

**Methods:**

A simulation framework for imaging near metal incorporating 3D implant and 3D human anatomical models, and imaging and sequence parameters is developed. MSI acquisitions are simulated based on susceptibility‐induced field shifts. Validation is performed using phantom and in vivo experiments at 0.55 and 3 T, comparing simulated and experimental images for artifact shape and SNR. Performance is demonstrated by evaluating impacts of field strength, spectral bins, RF and readout bandwidths, and implant materials.

**Results:**

Simulations accurately reproduced experimental imaging performance, with extent and patterns of metal artifacts closely matching experimental observations at both 0.55 and 3 T, while correctly predicting increased SNR and artifact size with increasing field strength. The framework demonstrated the impact of field strength, spectral bins, RF and readout bandwidths, and implant material on metal artifacts and image quality.

**Conclusion:**

This open‐source simulation framework effectively predicts MRI performance near metallic implants. This can facilitate protocol optimization and exploration of imaging parameters without extensive/costly in vivo experiments. This tool can enable researchers to predict imaging performance for various implants across different field strengths, potentially accelerating development of optimized protocols for clinical use.

## 1 Introduction

Orthopedic metallic implants are used in procedures such as joint replacements, spinal fusions, and fracture fixations, which are commonly used for the treatment of advanced osteoarthritis, degenerative disc disease, and fracture stabilization following trauma or resection. In the United States, approximately 7 million individuals were living with a hip or knee replacement in 2010, and this number is increasing each year [1]. There were approximately 2.5 million joint replacement surgeries performed from 2012 to 2021 [2], with total joint arthroplasty and spinal fusion among the most common procedures [3]. The increasing prevalence of metallic implants demonstrates the need for accurate non‐invasive imaging techniques that can capture the surrounding tissues, particularly those near or adjacent to the implants. This is essential for patient follow‐up and identification of possible complications, such as implant failure, dislocation, or infection.

The biggest challenge in magnetic resonance imaging (MRI) near metal is the susceptibility difference between metallic implants and human tissues, which causes large and rapidly varying magnetic field variations. Magnetic field variations change depending on the size, shape, type, orientation of the metal, and field strength [4]. Field inhomogeneity causes substantial artifacts such as signal loss due to signal dephasing or in‐plane and through‐plane distortions [5, 6]. Many variables can affect the metal artifact, such as implant geometry, field strength, imaging sequence, and sequence parameters. Techniques including view angle tilting (VAT) [7] and multi‐spectral imaging (MSI) can partially mitigate these effects and are now widely used at 1.5 T and 3 T. MSI techniques such as Slice Encoding for Metal Artifact Correction (SEMAC) [8], Multi‐Acquisition Variable Resonance Image Combination (MAVRIC) [9], and Multi‐Acquisition with Variable Resonance Image Combination Selective (MAVRIC‐SL) [10] correct artifacts by adding an extra encoding dimension to resolve off‐resonance spin locations.

Recent studies have shown promising results for imaging near metallic implants using contemporary low and mid‐field MRI systems. These systems demonstrate reduced susceptibility artifacts due to the lower field strength, potentially requiring fewer spectral encodings to achieve diagnostic image quality. Studies with hip implant phantoms at 0.55 T have shown significantly reduced metal artifacts compared to higher field strengths [11], with material‐dependent behavior showing titanium alloys producing the smallest artifacts [12]. Comparative studies between 0.55 T and higher fields have demonstrated that 0.55 T can provide equivalent or improved image quality near spinal hardware [13, 14]. A recent study comparing 0.55 T and 3 T MRI for patients with titanium total hip arthroplasty demonstrated significantly higher diagnostic confidence and reduced metal artifacts at lower field strength, even with conventional sequences [15].

This emerging trend toward lower field imaging requires comprehensive reoptimization of imaging protocols, including parameters such as RF excitation bandwidth, readout bandwidth, the number of spectral encodings, resolution, and SNR trade‐offs. However, optimizing these parameters through in vivo experiments can be time‐consuming and resource‐intensive and may expose patients to prolonged scanning sessions. Simulation frameworks offer an efficient alternative for exploring the parameter space and predicting imaging performance across different field strengths and implant materials. Such simulations can guide protocol development by identifying promising parameter combinations before experimental validation. This can potentially accelerate the clinical adoption of optimized low‐field MRI protocols for metallic implant imaging.

Simulations are incredibly valuable during the design and optimization of MRI methods, specifically for this application where the challenges of recruiting and scanning subjects with varying implant devices at varying field strengths make experimental optimization particularly difficult. One early work, presenting an integral method for numerical simulation of magnetic field perturbations induced by metallic objects represented as polyhedrons, demonstrated artifacts in spin‐echo images [16]. Another numerical model predicted magnetic field distortions and signal voids in gradient‐echo images near paramagnetic needle tips for various tip geometries and needle orientations [17]. The FORECAST method provided a fast‐Fourier‐transform approach to simulate off‐resonance artifacts in gradient‐echo sequences [18]. A simulation study characterized the limits of MRI near metallic implants in terms of RF excitation, water‐fat separation, and frequency encoding by analyzing field perturbations near digital implant models at 1.5 T and 3 T [19]. Recent numerical simulation approaches for predicting metal artifacts at different field strengths (1.5 T, 3 T, and 7 T) demonstrated similarity between simulated and measured artifact sizes for spin‐echo and gradient‐echo images with parameter exploration, including TE and bandwidth effects on artifact size [20, 21]. Recent work addressed the quantification of ripple artifacts in SEMAC sequence by using Bloch simulation for a digital implant inside a human model, analyzing how RF pulse parameters and slice thickness affect artifact appearance [22]. Simulations of the MAVRIC‐SL sequence were also used to validate approaches for susceptibility mapping around metallic implants and model‐based reconstruction for accelerated metal imaging [23, 24]. These simulation approaches collectively advanced our understanding of metal artifacts in specific contexts, such as sequence types, our ability to rapidly explore parameter spaces, and methods to validate different approaches.

In this paper, we demonstrate a framework for simulating MRI performance near metallic implants and provide an open‐source implementation. Building on prior models, we incorporate realistic 3D human anatomy with appropriate tissue properties, 3D metallic implant representation, and simulation of imaging near metal imaging techniques, including VAT, SEMAC, MAVRIC, and MAVRIC‐SL. The framework integrates susceptibility‐induced field perturbations, realistic noise modeling, tissue‐dependent parameters, and signal generation across different B0 field strengths. This simulation tool enables investigation of the effects of RF bandwidth, readout bandwidth, spectral encodings, and implant material on MRI without requiring extensive experimental studies, potentially facilitating protocol optimization for clinical applications. By providing an accessible tool, we aim to facilitate the broad development of optimized MRI around metal.

## 2 Methods

### 2.1 Simulation Framework

**FIGURE 1:**
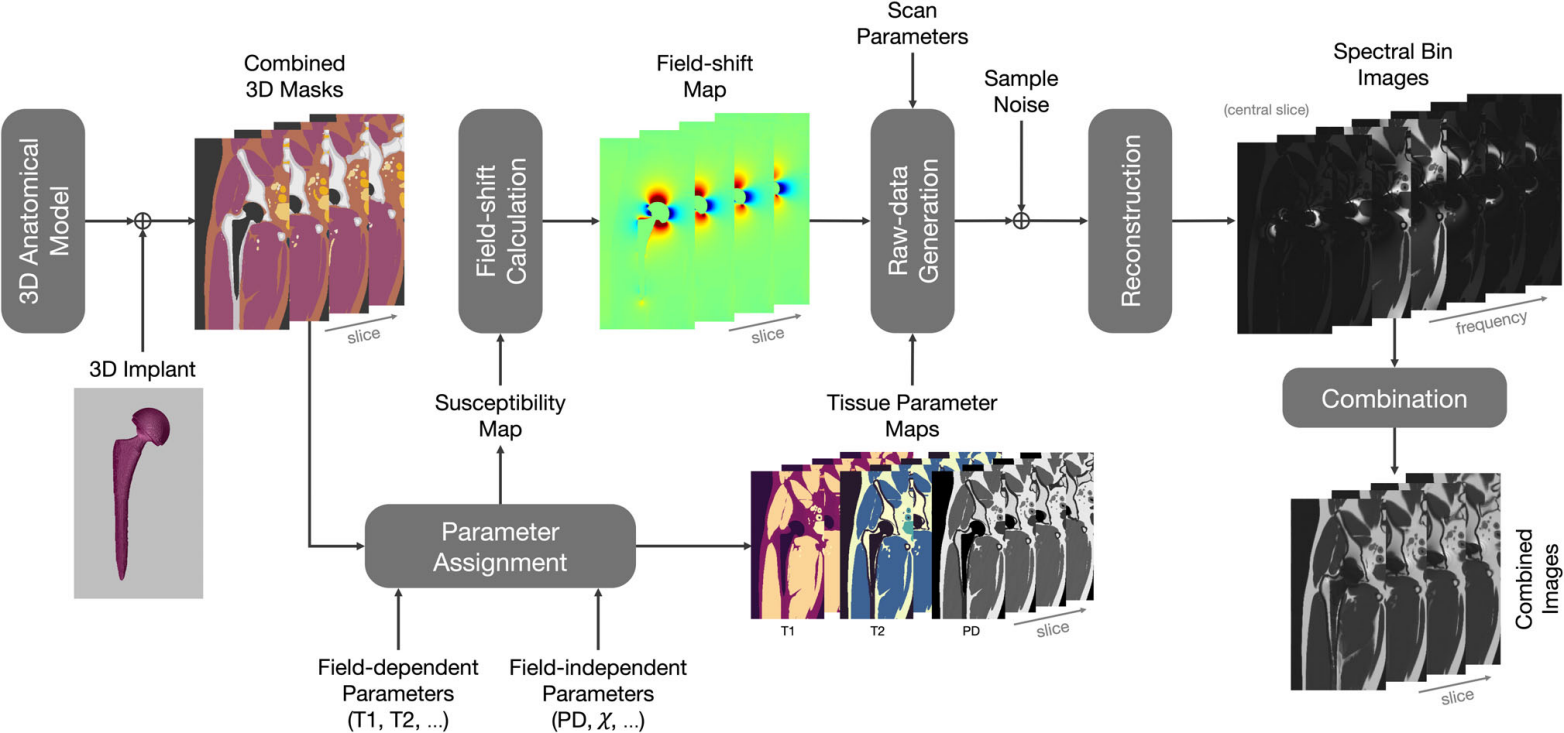
Simulation Pipeline. 3D body and hip implant masks are generated and utilized to assign parameters to tissues for susceptibility maps and tissue parameter maps. The susceptibility map that includes *χ* values for each implant material and tissue is used to calculate the susceptibility‐induced field shift map through Fourier‐based convolution. Raw data is simulated using the field shift map, tissue parameter maps, and sequence parameters. Complex Gaussian noise, scaled according to field strength, readout bandwidth, and voxel size, is added to simulate realistic SNR characteristics. Spectral bin images are reconstructed based on the imaging parameters provided as input and then combined with a root sum‐of‐squares summation.

Figure 1 illustrates the proposed simulation pipeline. Inputs fall into three categories: subject‐related, hardware‐related, and sequence‐related, and are listed in Table 1. The first step of the simulation is merging 3D anatomic masks with 3D implant masks. The resolution used for these masks should be finer than the imaging resolution in order to account for the intra‐voxel frequency distribution. Mask resolution is limited by computing power and memory. After merging, field‐dependent (T1, T2) and field‐independent (PD, *χ*) parameters are assigned to the combined masks to generate parameter maps. The *χ* map is then used to calculate the field shift (∆*f*) caused by the differences in magnetic susceptibilities.

**TABLE 1:** Inputs to the proposed simulator.

Subject‐related	Anatomy and Tissue properties	3D binary masks that correspond to biological tissues (e.g., muscle, bone, fat)
		T1, T2, PD, and magnetic susceptibility (*χ*) values for each biological tissue
	Implant geometry and composition	3D masks for each implant material
		Magnetic susceptibility (*χ*) values for each implant material
Hardware‐related	B0 field strength	
Software‐related	Sequence type (TSE, VAT, SEMAC, MAVRIC, MAVRIC‐SL)	
	Sequence parameters (TR, TE, RF bandwidth, readout bandwidth, imaging resolution, FOV, slice thickness, number of spectral encodings, number of averages)	

Simulated raw data is generated using the parameter maps and the spin echo signal equation $S=\rho \left(1-e^{-\mathrm{TR}/T_{1}}\right)e^{-\mathrm{TE}/T_{2}}$, which determines the image contrast. Excited spins are determined based on the RF excitation pulse shape, ∆*f* map, and slice gradient. The simulated raw data is based on collection of excited spins and frequency encode, phase encode, and slice gradients. For VAT acquisitions, slice gradients are played during readout. In the case of SEMAC acquisition, the raw data simulation step is repeated for each slice, and the central frequency of the slice is shifted in each step. SEMAC slice encodings are produced by applying z phase encode for each slice excitation produced with 2D sinc RF pulse. For MAVRIC and MAVRIC‐SL acquisitions, the raw data simulation process is repeated for each spectral bin image, and the central frequency of the bin is shifted in each step to generate multi‐spectral acquisition. Each spectral bin is created by using a 3D Gaussian RF pulse with a non‐selective and selective excitation, respectively for MAVRIC [9] and MAVRIC‐SL [10]. In the end, multi‐spectral raw data output is generated for both methods with different spectral encodings. Bivariate Gaussian noise is added to the simulated complex raw data independently based on the values of field strength, readout bandwidth, number of averages, and voxel size.

After raw data generation, spectral bin images are reconstructed with the imaging parameters provided as input. Multi‐channel receive is not implemented in this work, but could easily be incorporated by multiplying each spectral bin image with coil sensitivity maps. The combination of the spectral bin images is the final output of the simulation. In the case of SEMAC, the final image for each slice is obtained by combining spectral bin images for that slice, which are collected from slice encodings of other slice excitations. For MAVRIC and MAVRIC‐SL, the final image is obtained through a combination of excited volumes across the spectral dimension. Root sum‐of‐squares combination over the spectral dimension is performed for both methods for the final image.

### 2.2 Simulation Details

We utilized a 3D XCAT adult male body phantom as the input anatomy for the simulation [25]. A total hip replacement model with a femoral head and a femoral stem was 3D scanned by using Agisoft Metashape photogrammetry software [26], and the implant surface was coated with dry shampoo to avoid specular reflections from the metallic surface [27]. The enclosing volume of the 3D implant model was determined by using the alpha‐shape method [28]. Next, a 3D grid matrix of the same size as the input anatomy was created, and 3D masks for the implant model were obtained by checking whether a grid point was inside the enclosing volume or not. The accuracy of the resulting 3D implant model was validated by printing it with a 3D printer and comparing it with the actual implant visually, and quantitatively using calipers. Acetabular liners and cups were added to the implant by generating spherical shells of spherical caps. The 3D implant model was digitally inserted into the phantom anatomy, and 3D tissue masks were generated with isotropic resolutions. The simulated object was sampled at resolutions finer than the imaging resolution. Two primary sampling setups were used (0.2 × 0.2 × 0.2 mm and 0.5 × 0.5 × 0.5 mm resolutions), and an analysis with coarser and finer resolutions was performed to evaluate the impact of phantom resolution on simulation results.

Susceptibility values of the materials were set to 182 ppm for titanium, 900 ppm for cobalt‐chromium (CoCr), 2 ppm for ceramic, 9 ppm for polyethylene (PE), −9.05 ppm for tissues and water, −8.86 ppm for cortical bone, −5.55 ppm for fat, and 0.36 ppm for air [4, 5, 19, 29]. We assume that the induced magnetization of materials used in the digital phantom has a linear dependence on field strength. However, some metals like stainless steel can have a nonlinear magnetization behavior that saturates with the increased field strength [30]. The susceptibility‐induced field shift was calculated using a Fourier‐based convolution method [18, 31]. Proton density values of the tissues including muscle, fat, bone, bone marrow, cartilage, and blood were determined based on an in vivo PD‐weighted scan. Field‐dependent T1 values of the tissues were calculated by fitting an exponential tissue dispersion model to the previous relaxation parameter values reported in the literature [32, 33, 34, 35, 36, 37, 38, 39, 40]. We assumed that T2 values do not vary significantly with field strength and assigned constant values to these tissues based on earlier values reported at 0.55 T [36, 37]. However, the framework allows users to define their own model or input other T1 and T2 values.

Noise calibration was performed using an in vivo 0.55 T scan from a healthy adult volunteer and adapted and scaled based on the input parameters. Additive bivariate complex‐valued Gaussian noise data was added to synthesized raw data to match the SNR of the thigh muscle closest to the femur bone of the 0.55 T data and body noise dominance was assumed. Noise standard deviation was scaled with $\left(B_{0}/B_{0,\mathrm{ref}}\right)\left(V_{\mathrm{ref}}/V\right)\sqrt{\mathrm{BW}/{\mathrm{BW}}_{\mathrm{ref}}}\sqrt{{\mathrm{NEX}}_{\mathrm{ref}}/\mathrm{NEX}}$ and signal strength was scaled with ${\left(B_{0}/B_{0,\mathrm{ref}}\right)}^{2}$ where subscript ref represents the reference acquisition, BW readout bandwidth, NEX number of averages, and V voxel volume.

### 2.3 Validation

Experiments were performed on phantoms and adult volunteers. 0.55 T scans were performed on a whole‐body 0.55 T scanner (prototype MAGNETOM Aera, Siemens Healthineers, Forchheim, Germany) equipped with high‐performance shielded gradients (45 mT/m amplitude, 200 T/m/s slew rate). 3 T scans were performed on a whole‐body 3 T scanner (MAGNETOM Prisma Trio, Siemens Healthineers, Forchheim, Germany) equipped with high‐performance shielded gradients (80 mT/m amplitude, 200 T/m/s slew rate). All subjects provided written informed consent and were scanned under a protocol approved by the Institutional Review Board.

Phantom experiments described here were performed to validate the simulator's ability to capture the dependence of metal artifacts on imaging parameters. A 3D total hip replacement with a cobalt‐chromium head and a titanium stem placed inside a water bath was scanned with SEMAC at 0.55 T. This model was also 3D scanned using photogrammetry and digitally placed into a simulated water bath of approximately the same size as used experimentally. The simulation was run with imaging and sequence parameters identical to the actual scan (except for a minor difference in the RF envelope shape, which had the same bandwidth). The simulation had 2 × 2 × 8 times finer sampling resolution (0.5 × 0.5 × 0.5 mm) than the imaging resolution (1 × 1 × 4 mm). Acquisition parameters were: TR = 2550 ms, TE = 37 ms, FOV = 260 × 320 mm, resolution = 1 × 1 mm, slice thickness = 4 mm, readout bandwidth = 600 Hz/Px, RF bandwidth = 1 kHz, VAT = 100%, SEMAC factor = 6 and 12, superior–inferior (SI) frequency encoding direction, and coronal orientation. Note that the VAT percentage corresponds to the percentage of the slice gradient played during the readout. The shape and size of the metal artifacts and spectral bin images were qualitatively compared to test whether the simulation matched experimental imaging performance.

An in vivo experiment was performed to validate the simulator's ability to capture artifact dependence on B0 field strength and imaging parameters, and its ability to reflect a realistic anatomy. One volunteer with a total hip replacement (Titanium acetabular cup, PE liner, CoCr femoral head, Titanium femoral stem) was scanned at 0.55 T and 3 T. Data were collected using a 6‐channel body coil wrapped around the hip. Acquisition parameters at 0.55 T were: TR = 2100 ms, TE = 31 ms, FOV = 280 × 228 mm, resolution = 0.9 × 0.9 mm, slice thickness = 4 mm, readout bandwidth = 401 Hz/Px, RF bandwidth = 1 kHz, VAT = 100%, SEMAC factor = 12, SI frequency encoding direction, and coronal orientation. Acquisition parameters at 3 T were: TR = 4050 ms, TE = 32 ms, FOV = 280 × 280 mm, resolution = 0.9 × 0.9 mm, slice thickness = 3 mm, readout bandwidth = 710 Hz/Px, RF bandwidth = 1 kHz, VAT = 100%, SEMAC factor = 20, SI frequency encoding direction, and coronal orientation. The same anatomical region integrated with a total hip replacement implant was simulated with the same imaging parameters (except RF pulse shape) and the same implant materials. The simulation had a sampling resolution of 0.2 × 0.2 × 0.2 mm. The shape of the metal artifacts and anatomy were qualitatively compared to test whether the simulation matched experimental imaging performance at the two field strengths.

### 2.4 Quantitative Performance Evaluation

To evaluate the images quantitatively, we measured the length and area of the signal void and compared them with the length and area of the implant, as shown in Figure S1. To determine the signal void area, we performed additional simulations with identical parameters but with implant susceptibility set to match human tissue, then calculated the difference between the final images with and without metal susceptibility. The signal void area was determined by manually tracing the outline of the signal void area in a central slice from the difference image and calculating the area inside the outline. The signal void length was measured as the maximum extent of the signal void in the right–left (RL) and superior–inferior (SI) directions. SNR was calculated by dividing the mean signal intensity in a region of interest (ROI) placed in the muscle tissue by the standard deviation of the noise in a background ROI. SNR calculations were performed on magnitude images, and the background noise statistics were corrected for the Rayleigh distribution of magnitude noise. All simulations and analyses were performed in MATLAB R2021a (The MathWorks Inc., MA, USA). Simulation time was measured in a workstation with 32‐core CPU and 4 TB RAM.

### 2.5 Demonstration

We next demonstrated the functionalities of this framework to predict performance for various scenarios. We performed simulated parameter sweeps, where we investigated the impact of parameters on metal artifacts and image quality. Simulations were performed with the human anatomic model combined with the 3D total hip replacement model (CoCr cup, PE liner, CoCr head, and Titanium stem). The following simulation parameters were used as a baseline for the parameter sweeps: TR = 2000 ms, TE = 34 ms, FOV = 400 × 200 mm, resolution = 1 × 1 mm, slice thickness = 3 mm, readout bandwidth = 400 Hz/Px, RF bandwidth = 1 kHz, number of spectral encodings (SEMAC factor) = 4, 6, 12, 18, and 24, SI frequency encoding direction, and coronal orientation.

We examined the impact of B0 field strength (0.05 T, 0.1 T, 0.2 T, 0.3 T, 0.55 T, 1 T, 1.5 T, 3 T, and 5 T) on the appearance of metal artifacts. All simulation parameters were kept the same while field strength was varied. We also examined the impact of sequence parameters. We explored the use of different numbers of spectral encodings (4, 6, 12, 18, 24) for each field strength. We tested different RF bandwidths (0.5 kHz, 0.75 kHz, 1 kHz, 1.25 kHz, 1.5 kHz) at 0.55 T for SEMAC with 6 spectral encodings. We demonstrated the impact of readout bandwidth (100 Hz/Px, 200 Hz/Px, 400 Hz/Px, 600 Hz/Px and 800 Hz/Px) on in‐plane artifacts and their corrections at 0.55 T by comparing TSE and VAT sequences.

We examined the effect of implant material types on metal artifacts by simulating two different total hip replacement head configurations at varying field strengths: CoCr cup, PE liner, and CoCr head with SEMAC factor of 12, and Titanium cup, PE liner, and ceramic head with SEMAC factor of 6.

We performed an analysis of signal contributions from individual spectral bins to provide guidance for determining the minimum number of spectral encodings for the two specific implant configurations (CoCr and Titanium). We simulated SEMAC with 36 spectral encodings to comprehensively cover the spectral distribution at 0.55 T, 1.5 T, and 3 T for both implant configurations using noiseless simulations with only the implant model (without the digital human). We calculated the percentage of signal within 3 cm of the implant boundary for each spectral bin to quantify the contribution of each bin to the final image.

A grid sensitivity analysis was performed using phantom resolutions of 1, 0.5, 0.2, and 0.1 mm isotropic (corresponding to 1 × 1 × 3, 2 × 2 × 6, 5 × 5 × 15, and 10 × 10 × 30 times finer than imaging resolution, respectively) for SEMAC simulations with 18 spectral encodings at 0.55, 1.5, and 3 T.

For all parameter studies, only the variable of interest was changed while other parameters remained constant. The simulation for the effect of the field strength and implant material demonstrations had 5 × 5 × 15 times finer sampling resolution than the imaging resolution, while all other simulations used the phantom with 2 × 2 × 6 times finer sampling resolution.

## 3 Results

### 3.1 Validation

**FIGURE 2:**
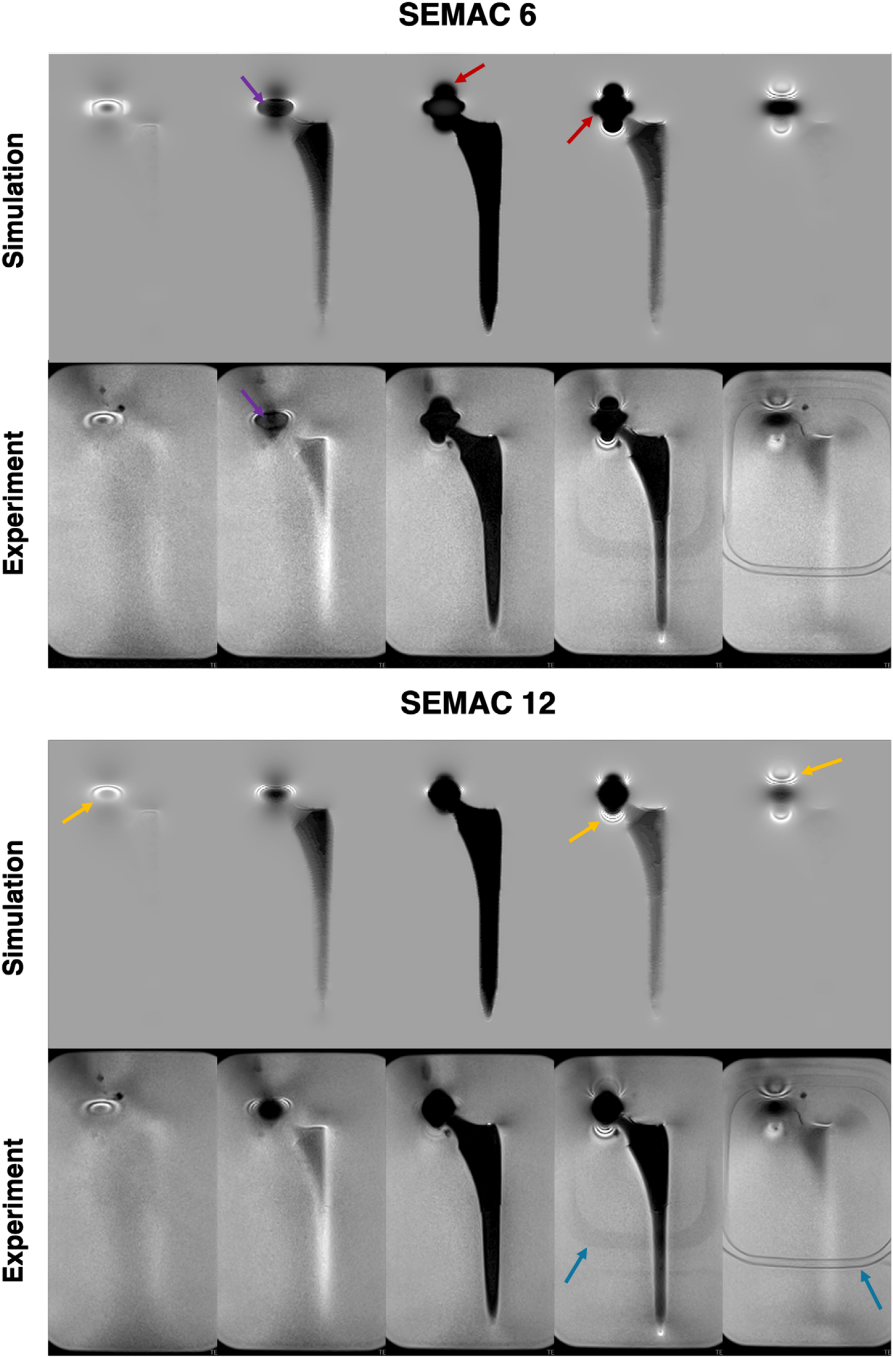
Validation of simulation using a THA phantom and SEMAC reconstruction. Shown is a comparison of experiment (top row) and simulation (bottom row) with identical parameters, for total hip replacement hardware with a CoCr head and Titanium stem in a water bath at 0.55 T. The simulation accurately reproduces experimental SEMAC imaging artifact patterns, including signal void regions (red arrows), ripple artifacts (orange arrows), and aliasing due to undersampling in the spectral encoding dimension (purple arrows). Note the close correspondence in artifact shape, extent, and signal characteristics. Subtle image differences are due to (1) difficulty matching the exact orientation, placement, and geometry of the implant, and (2) the use of a plastic lid to hold the implant (visible in some experiment slices, blue arrows).

**FIGURE 3:**
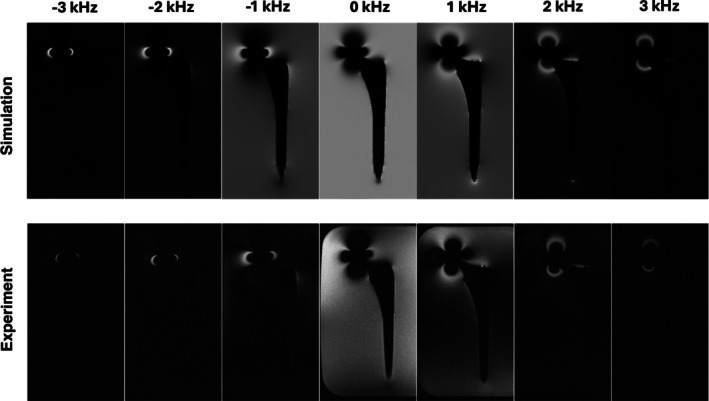
Validation of simulation using a THA phantom and SEMAC spectral bin images. Spectral bin images for experiment (top row) and simulation (bottom row) with identical parameters that match the middle slice in Figure 2. Qualitatively, the simulation reproduces the experimental spectral distribution patterns around the implant very well. Subtle differences in spectral distributions are due to differences in RF pulse shapes. Experiments used the vendor's proprietary excitation waveform, and simulations used an in‐house generated waveform (windowed sinc).

Figure 2 shows a comparison between the experimental and simulated SEMAC images of the total hip replacement placed into a water bath at 0.55 T. The size and shape of the artifacts are found to be very similar in both images. This indicates that the simulation accurately reproduced the imaging performance of the experimental setup. A qualitative comparison of the spectral bin images is shown in Figure 3. The simulated bin images matched the experimental bin images, with similar spectral bin patterns observed in both. These results suggest that the simulation can be a useful tool to predict metal artifacts and multi‐spectral imaging performance and optimize protocols for imaging near metallic implants.

**FIGURE 4:**
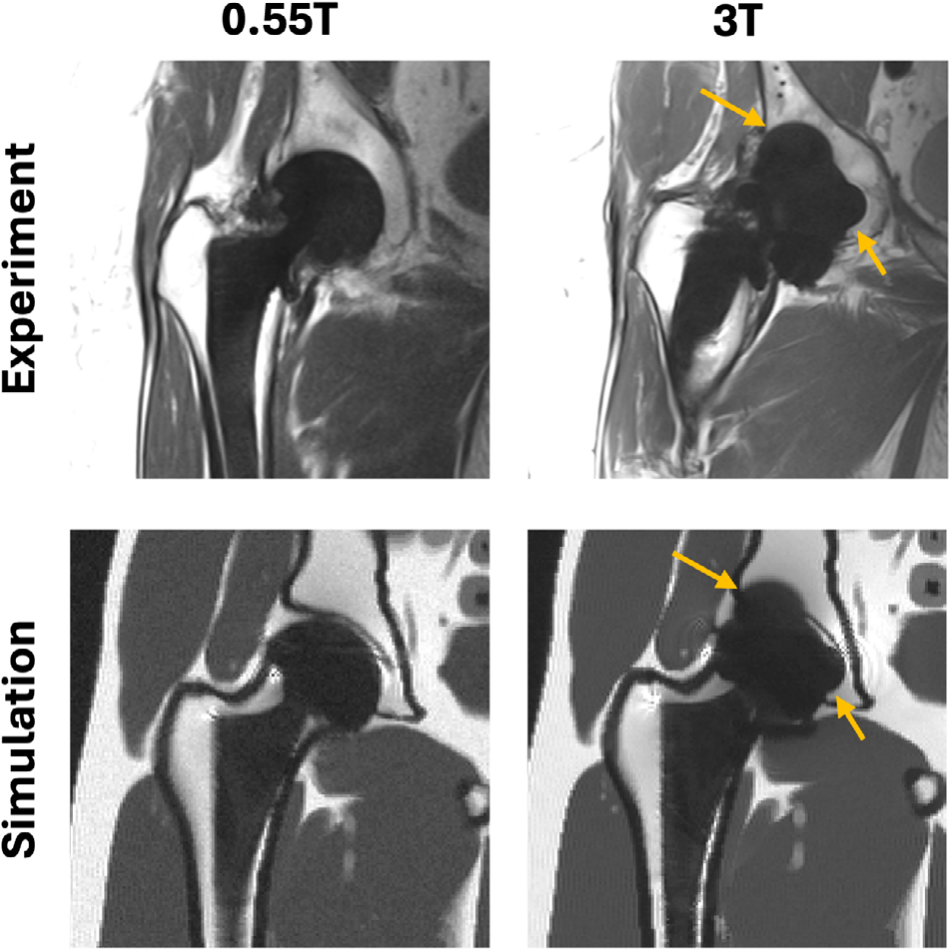
Experimental qualitative validation in a patient with total hip replacement. Shown are experiment (top row) and simulation (bottom row) at both 0.55 and 3 T. The simulation captures the overall trend of increased artifact size at higher field strength, and several key features such as increased signal void area and reduced tissue visibility near the implant at 3 T compared to 0.55 T (orange arrows). This is observed despite using a generic anatomical model. There are subtle differences in artifact patterns due to the implant configuration not being an exact match to the volunteer.

Figure 4 shows the qualitative comparison of metal artifact shapes and anatomy between the experimental scans and the simulations at 0.55 and 3 T. The metal artifact trends from 0.55 to 3 T in the simulated SEMAC images match those observed in the volunteer scan images for both field strengths. These results suggest that the proposed simulation can be a valuable tool for estimating the artifact shapes at different field strengths and planning the scan protocols for patients with metal implants accordingly.

### 3.2 Demonstration

**FIGURE 5:**
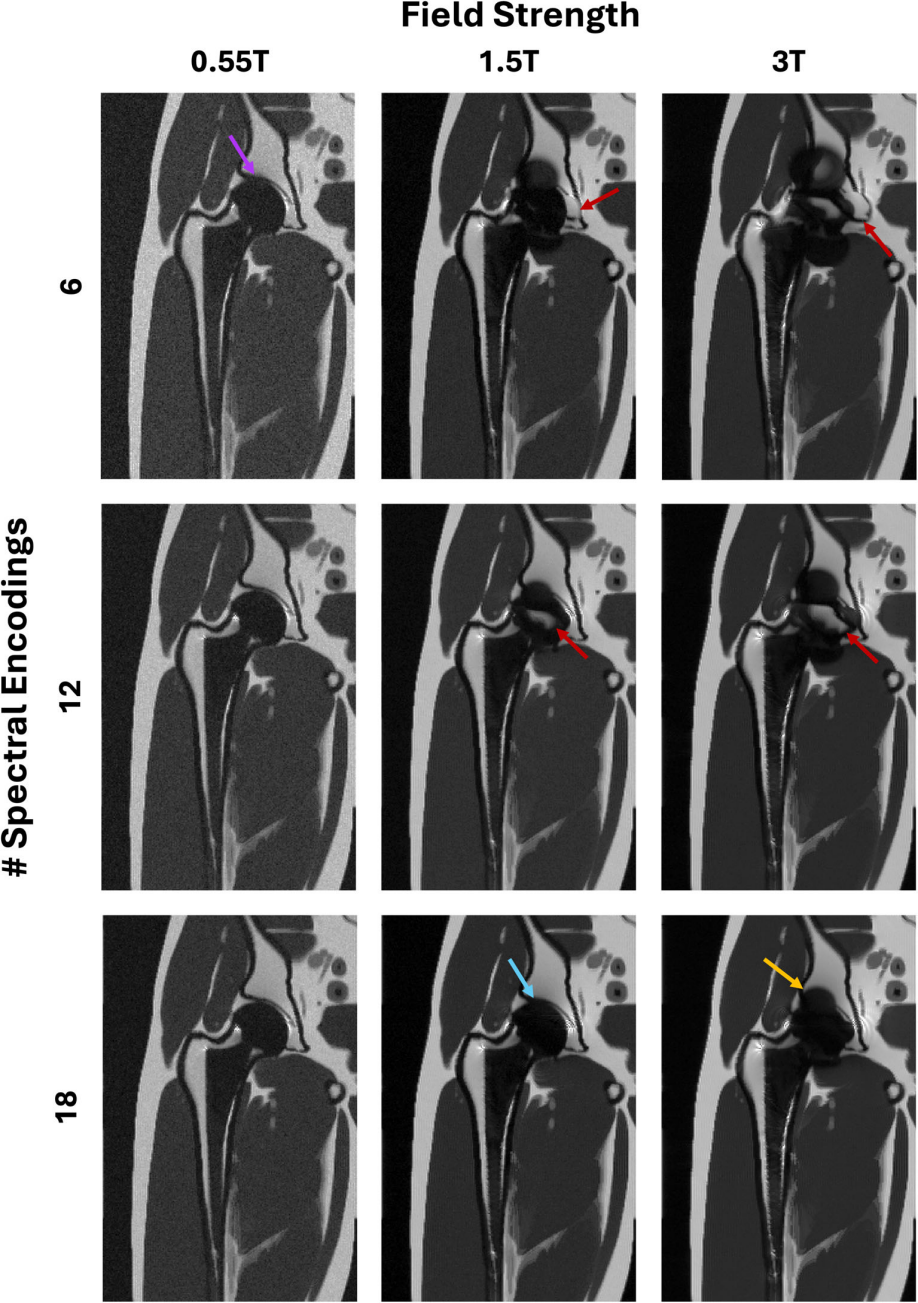
Illustration of SNR and artifact dependence on B0 field strength (horizontal axis) and SEMAC factor (vertical axis). As field strength increases from 0.55 to 3 T, both SNR and the signal void area increase substantially. The number of spectral encodings significantly affects artifact reduction, with higher factors providing better correction at the cost of longer scan times (not listed). At 0.55 T, even with relatively few spectral encodings (6), artifacts remain close to the implant (purple arrow), while at 1.5 T, more spectral encodings (18) are required to achieve comparable artifact reduction (blue arrow). At 3 T, the maximum number of spectral encodings simulated (18) is not sufficient to achieve the same level of artifact reduction observed (orange arrow). Aliasing artifacts inside the signal void regions are also seen in lower spectral encodes (6, 12) at 1.5 and 3 T due to undersampling in the spectral encoding dimension (red arrows).

Figure 5 shows the impact of field strength (0.55 T, 1.5 T, and 3 T) on the appearance of metal artifacts for SEMAC scans with different numbers of spectral bins. The figure illustrates that the shape of the metal artifact gets larger as the field strength increases. The number of spectral bins also has a significant impact on the metal artifact, with fewer spectral bins leading to more artifacts, such as larger signal voids and aliasing due to undersampled spectral encoding dimension. At lower field strength (0.55 T), the artifacts near the implant are very close to the implant head, especially for high numbers of spectral bins, whereas the artifact starts to cause a substantial distortion near the surrounding tissues when field strength increases.

**FIGURE 6:**
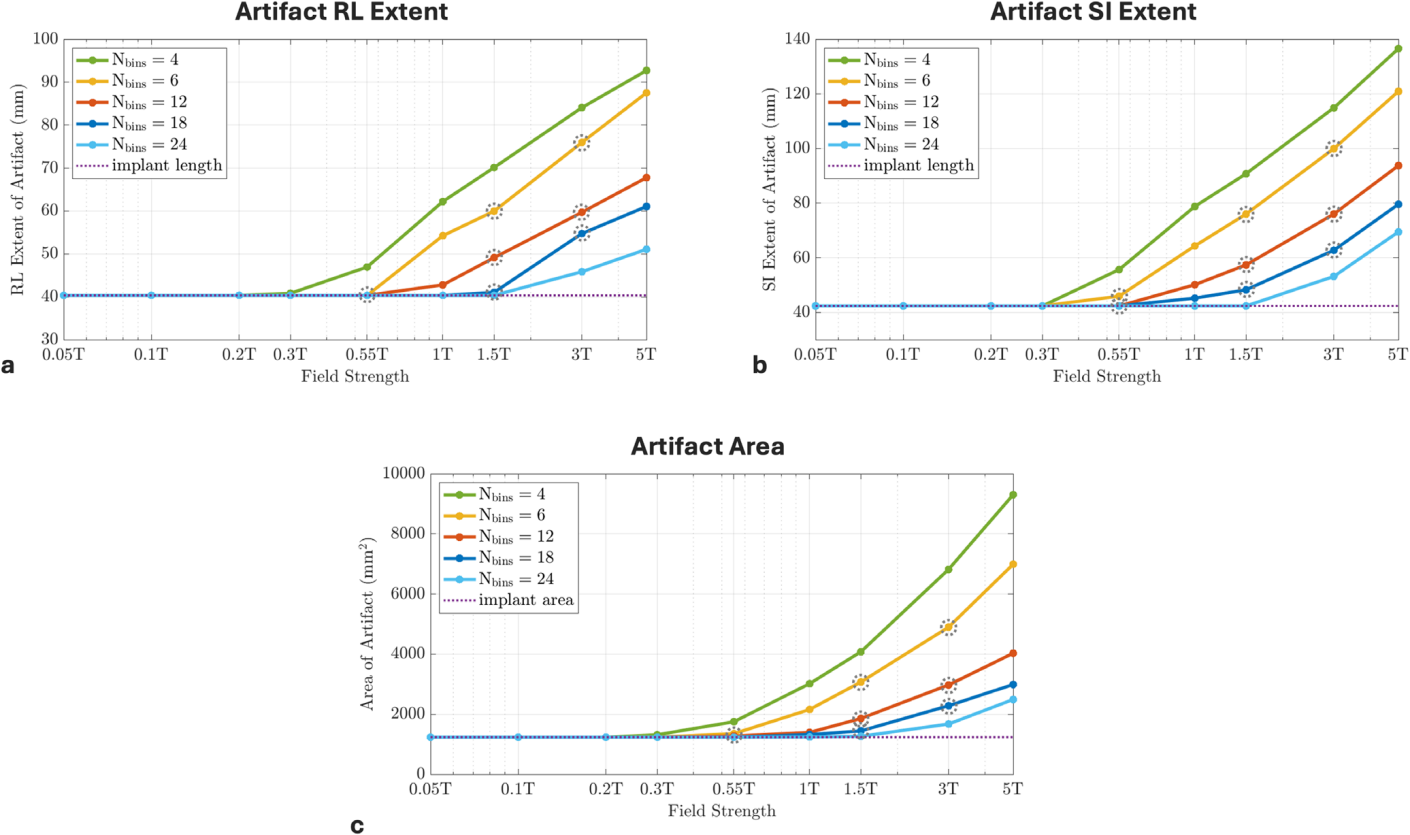
Quantitative metrics as a function of B0 field strength. Shown are (a) signal void artifact right–left extent (mm), (b) artifact superior–inferior extent (mm), and (c) artifact area (mm^2^) plotted against field strength with separate lines for different spectral encoding factors. Field strength values are plotted on a logarithmic scale from 0.05 to 5 T. Measurements corresponding to the images in Figure **5** are marked with gray dashed circles. Implant lengths and implant area are shown as dashed purple lines in (a–c) to provide a baseline reference. The extent and area of the artifact are greatly reduced at lower field strengths. At lower field strengths, a closer area around the implant can be captured with fewer spectral encodings compared to higher fields (e.g., *N*_bins_ = 6 at 0.55 T provides artifact reduction comparable to *N*_bins_ = 18 at 1.5 T). Signal void artifacts are not observed at field strengths below 0.2 T with this simulation setup.

Figure 6 provides plots for quantitative metrics calculated from simulations performed at various field strengths using different spectral encodings, such as signal void artifact right–left extent (mm), superior–inferior extent (mm), and artifact area (mm^2^) as a function of B0. These plots demonstrate that the artifact extents and artifact area are almost proportional to the field strength. Notably, at 0.55 T with 12 spectral bins, the artifact area is only 3% larger than the actual implant head area, while at 3 T with the same number of bins, the artifact area is nearly 140% times larger than the actual implant head area. Additionally, no signal void artifacts are detected at field strengths below 0.2 T using this simulation setup. This quantitative analysis confirms the significant advantages of lower field strengths for metal artifact reduction.

**FIGURE 7:**
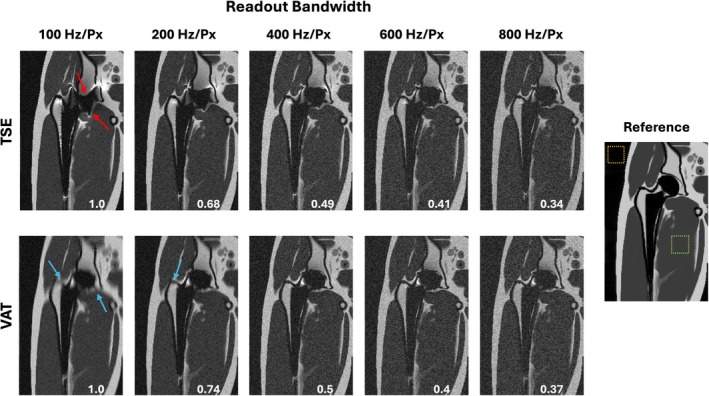
Illustration of dependence of artifact on readout bandwidth at 0.55 T for TSE (top row) and VAT (bottom row). TSE and VAT simulations across a range of readout bandwidths (100, 200, 400, 600, and 800 Hz/Px) while the other parameters fixed. Relative SNR values between images are written at the bottom of each image. SNR is measured from the muscle and noise ROIs (green and yellow dashed boxes shown on the noiseless reference image on the right). As readout bandwidth decreases, in‐plane distortions increase in TSE images due to larger phase accumulation (red arrows). VAT effectively corrects these in‐plane distortions across all bandwidth settings by compensating for in‐plane frequency shifts, but introduces blurring at lower bandwidths (blue arrows). SNR increases with a decrease in readout bandwidth, as is evident in both sequence types. This demonstrates the importance of selecting readout bandwidth for the best compromise between in‐plane artifact correction, potential blurring, and image quality when imaging near metal.

Figure 7 illustrates the effect of readout bandwidth on artifact and image quality at 0.55 T. Simulations compare conventional TSE and VAT using a range of readout bandwidth per pixel from 100 to 800 Hz/Px. The figure demonstrates that as readout bandwidth decreases, in‐plane distortions increase in TSE due to larger frequency‐induced phase accumulation. VAT effectively corrects these in‐plane distortions across all bandwidth settings but introduces some blurring at lower bandwidths. The advantage of lower readout bandwidth is increased SNR, which is evident in both sequence types, presenting a trade‐off between artifact correction and image quality.

**FIGURE 8:**
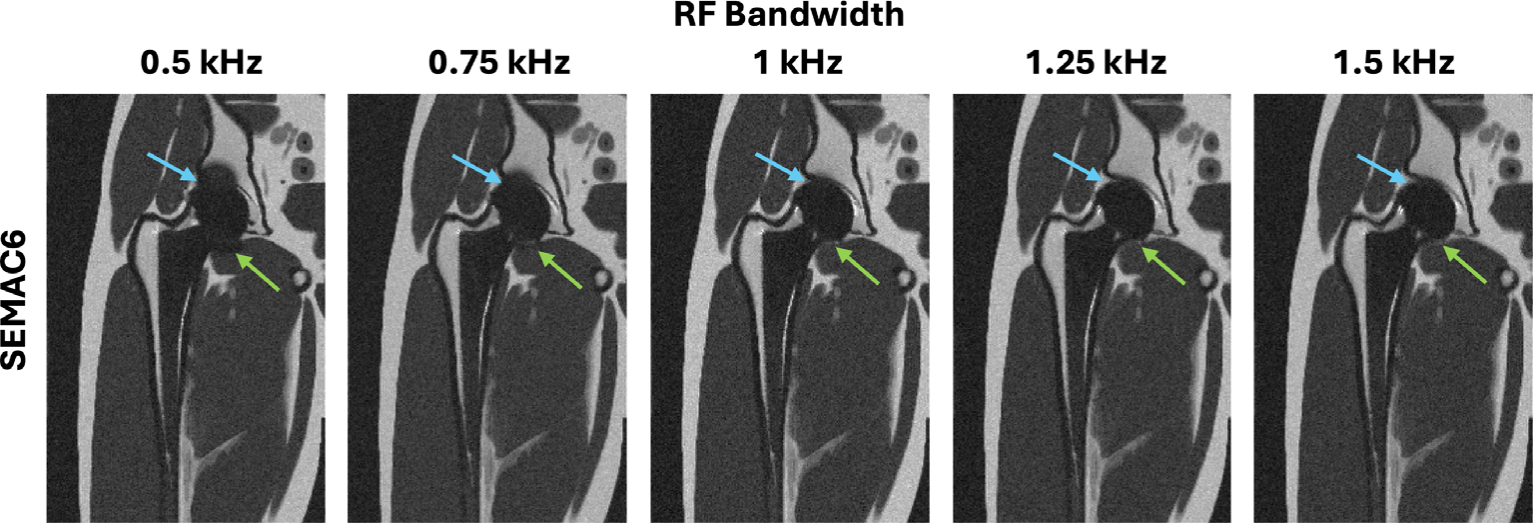
Illustration of dependence of artifact on RF bandwidth at 0.55 T for SEMAC. SEMAC simulations with six spectral encodings across a range of RF bandwidths while the other parameters fixed. As RF bandwidth increases, the same number of spectral encodings provides improved coverage of the frequency distribution near the implant. This allows better signal recovery in regions closer to the implant (blue and green arrows).

Figure 8 shows the impact of RF bandwidth on SEMAC performance with 6 spectral encodings at 0.55 T. Simulations are shown at a range of RF bandwidths from 0.5 to 1.5 kHz with a fixed readout bandwidth of 400 Hz/Px. The figure illustrates that as RF bandwidth increases, it results in better signal recovery near the implant. Wider RF bandwidths lead to improved coverage of the frequency distribution, thereby recovering more signals closer to the implant which typically correspond to higher off‐resonance frequencies.

Figure S2 illustrates the effect of implant material on metal artifacts. The figure compares the appearance of simulated metal artifacts between two different implant configurations: one with CoCr components using SEMAC 12 and another with Titanium/ceramic components using SEMAC 6. Despite the titanium setup using fewer spectral encodings, it produces substantially smaller artifacts than the CoCr implant at all field strengths (0.55 T, 1.5 T, and 3 T). The difference between materials becomes increasingly pronounced at higher field strengths, with 3 T showing the largest disparity in artifact size between the two implant configurations.

Figure S3 provides the grid sensitivity analysis results. No significant differences are observed between images simulated with 0.1 and 0.2 mm isotropic phantom resolutions across all field strengths tested. Differences become apparent at 0.5 mm resolution, particularly at object boundaries, likely due to discrete object approximation affecting off‐resonance calculations at implant edges. The 1 mm resolution shows substantial discretization artifacts and is generally insufficient for accurate simulation outcomes. The impact of discretization becomes more pronounced at higher field strengths, where off‐resonance effects are proportionally larger.

Figures S4–S6 present the analysis of signal contributions from individual spectral bins for the specific THA implant configuration studied. Figure S4 shows bar plots of signal percentages within 3 cm of the implant boundary for each spectral bin, comparing CoCr and Titanium configurations across field strengths. Figure S5 displays individual spectral bin images for the CoCr configuration, while Figure S6 shows the corresponding images for the Titanium configuration. For both implant configurations, the analysis demonstrates that at lower field strengths, signal is concentrated in a narrow spectral range, while at higher field strengths, the signal distribution becomes broader and more dispersed. The Titanium configuration shows a more concentrated spectral distribution due to lower magnetic susceptibility, requiring fewer spectral encodings compared to the CoCr configuration to capture the same percentage of recoverable signal at all field strengths demonstrated. The signal spatially closest to the implant is recovered in the furthest spectral bins from the central bin, although these bins contribute lower signal percentages. These findings are specific to this implant geometry and simulation setup, and similar analyses should be performed for different implant configurations.

Simulation times varied with implant sampling resolution and sequence. For TSE and VAT sequences using the isotropic 0.5 mm phantom setup, the average simulation time was 8 min and 48 s. For SEMAC sequences with the 0.5 mm phantom setup, simulation times were approximately 47 min, 1 h and 29 min, and 2 h and 16 min for 6, 12, and 18 spectral encodings, respectively. SEMAC simulations using the phantom with finer sampling (0.2 mm) required longer times: approximately 4 h and 22 min, 6 h and 40 min, and 10 h and 16 min for 6, 12, and 18 spectral encodings, respectively.

## 4 Discussion

Recent studies have shown that MRI at low field strengths is promising for imaging near metal, providing reduced distortions and signal voids [11]. The proposed simulator accurately portrays the field‐strength dependence of metal artifacts. Therefore, it can also be used to explore what may be possible at field strengths that have not yet been physically realized, providing valuable insight.

This work can be generalized to other human body models. We incorporated one realistic adult male body model to provide a better depiction of the impact of the metallic artifacts on the tissues near the implant. Other human body models that are available in voxelized format or can be converted to voxelized format are compatible with the simulator. There are several families of computational phantoms available [41], such as anatomically variable XCAT phantom variants [42, 43, 44], UF phantoms [45], and the Virtual Population (ViP) phantoms [46], each with an associated cost. It is worth noting that these models may not represent the full range of abnormalities, for example, major spine deformities or pathological tissue variations. In this work, we did not model tissue heterogeneity within organs, as this information was not included in the phantom we used. This is noticeable from the relatively homogeneous appearance of subcutaneous fat and muscle tissues in our simulations compared to actual patient images in Figure 4.

Accurate simulations require detailed technical specifications, including 3D geometric models of implant components, complete material compositions, and their corresponding magnetic susceptibility values. There are many ways to obtain the 3D implant model. In our work, we used photogrammetry where the implant is created using multiple photos of the implant taken from different angles. Photogrammetry is widely accessible; however, it has limitations. The quality of the resulting model can be affected by lighting conditions, reflectiveness and shininess of the object, and the accuracy of the image alignments [27]. One could also use CAD models provided by vendors [19] or generated by 3D scanners [47] or reconstruct implant models based on CT scans [23, 24]. We also manually generated acetabular cup geometries and obtained magnetic susceptibility values from published literature. However, direct access to manufacturer specifications would significantly improve simulation accuracy and enable more widespread use of simulation‐based protocol optimization.

Manual placement of the implant in the human model is time‐consuming (e.g., several hours). Placement may take even more time in complicated situations with multiple interconnected components such as multilevel spinal fusion. Accurate placement of the implant also requires knowledge of the anatomy and surgical technique. Software tools for time‐efficient placement of implants may help reduce the time required.

The simulator itself has several limitations. We did not include the impact of eddy currents, gradient nonlinearity, concomitant fields, or other encoding imperfections. These were omitted because, in general, the effects are less significant than the metal‐induced susceptibility artifacts, and their inclusion would be computationally expensive. Future work could address these limitations by using a full Bloch simulation. This study only considered a single‐body coil; however, it can be easily extended to accommodate multi‐coil scenarios.

We modeled field‐dependent T1 values using a simple power‐law relationship (T1 = A × B_0_^B^), where parameters A and B were determined by fitting to published relaxation values [32, 33, 34, 35, 36, 37, 38, 39, 40]. We also assumed that T2 values do not vary significantly with field strength and assigned constant values to these tissues based on values reported in the literature. However, the framework allows users to define their own model or input other T1 and T2 values. While this approach provides reasonable approximations, more sophisticated models with more sample relaxation values across various field strengths could improve simulation accuracy, especially when exploring imaging at a non‐conventional field strength.

We made several approximations in order to control computational expense. Since there is no signal displacement along the phase encoding direction, phase accumulation along the phase encoding direction is ignored. This is only applicable to Cartesian imaging, and will require modification in order to be applicable to other sampling schemes. Additionally, for most simulations, we used a relatively low (2 × 2 × 6) tissue oversampling factor to represent intravoxel frequency variations. Simulations for field strength and implant material comparisons used higher oversampling factors of 5 × 5 × 15 times finer sampling resolution than the imaging resolution. Simulations using higher oversampling factors are more accurate, especially at higher field strengths where there are larger intravoxel frequency variations, but more computationally expensive. Grid sensitivity varies with material composition, implant geometry, and orientation, requiring users to perform sensitivity analyses for their specific configurations.

Simulation times can be further reduced through several strategies: (1) optimizing grid oversampling factors based on sensitivity analysis for the specific scenario, as unnecessarily high oversampling provides little improvement in accuracy while substantially increasing computation time; (2) improving implementation efficiency through parallel processing of slices and/or spectral bins, migration to faster languages such as C++ or Python, use of optimized libraries, or GPU acceleration; and (3) leveraging continuing improvements in computational hardware performance.

The proposed framework includes implementations of MAVRIC and MAVRIC‐SL sequences in addition to SEMAC. However, we chose not to present simulation results for these sequences in this paper, as we currently do not have the ability to validate them. The codebase contains the implementation of these sequences, allowing users to simulate them. Further experimental validation would be needed to assess the accuracy of these implementations across different field strengths and implant types. Such validation would enable more comprehensive comparisons of various MSI techniques for different scenarios.

Our quantitative evaluation of metal artifacts is limited to basic metrics such as artifact area and extent. A more comprehensive assessment could be achieved by incorporating the recently proposed nonuniform image quality framework for imaging near metal [47]. Future integration of this systematic approach into our simulation framework would enable a more detailed evaluation of protocol optimization parameters by quantifying various image quality metrics in different regions surrounding the implant.

## 5 Conclusion

We demonstrate a simulation framework that accurately reproduces MRI signal behavior and distortions in the presence of metallic implants, including the dependence on imaging parameters and field strength. This can be used for time‐efficient MRI protocol optimization for patients with metallic implants, while also providing the ability to realistically explore the impact of field strength, implant material and composition, and imaging parameters without requiring experiments with human subjects. As metallic implants are diverse in shape and material composition, in vivo optimization is often impractical as it requires imaging large numbers of subjects with time‐consuming protocols.

## Supporting information


**Figure S1:** Quantitative evaluation of the artifact. (Left) Simulation with metal susceptibility showing the signal void artifact around the implant. (Middle) Simulation with identical parameters but implant susceptibility set to match human tissue, showing the expected signal without metal‐induced artifacts. (Right) Absolute difference image obtained by subtracting the tissue‐matched simulation from the metal simulation, emphasizing the artifact region appearing with intensities corresponding to the underlying tissue values. The positive signal visible at the location of the metal implant (indicated by purple arrow) is due to undersampling in the spectral domain, which causes aliasing artifacts along the spectral dimension. Artifact right–left extent and superior–inferior extent measure the maximum horizontal and vertical dimensions of the signal void, while artifact area quantifies the total two‐dimensional area affected by the signal loss. The measurement regions for area (red overlay) and length extents (yellow and blue dashed lines) are overlaid on the difference image. Artifact measurements were performed by manual tracing of visually apparent signal void boundaries in the difference images. These measurements are compared against the actual implant dimensions to determine the degree of artifact sizes at different field strengths and sequence parameters.
**Figure S2:** Illustration of the effect of implant material on artifact size at 0.55, 1.5, and 3 T. Comparison of metal artifacts between two different implant head configurations: one with CoCr components using SEMAC 12 and another with Titanium/ceramic components using SEMAC 6. Despite the Titanium setup using fewer spectral encodings, it consistently produces smaller artifacts than the CoCr implant at all field strengths due to its lower magnetic susceptibility, and the artifacts are predominantly localized around the neck of the implant (orange arrows). The difference between materials becomes more pronounced at higher fields, with 3 T showing the largest disparity in artifact size. This demonstrates that lower susceptibility materials allow for fewer spectral encodings and, therefore, shorter scan times.
**Figure S3:** Impact of phantom resolution on simulation accuracy. (a) Simulation of SEMAC images with 18 spectral encodings using phantom resolutions of 1, 0.5, 0.2, and 0.1 mm isotropic (1 × 1 × 3, 2 × 2 × 6, 5 × 5 × 15, and 10 × 10 × 30 times finer than imaging resolution, respectively) at field strengths of 0.55, 1.5, and 3 T. (b) Absolute differences between the finest resolution (0.1 mm) and other resolutions (1 mm, 0.5 mm, and 0.2 mm) (shown with 5× enhancement) to identify regions where phantom resolution significantly impacts simulation results at each field strength. No significant differences are observed between images simulated with 0.1 and 0.2 mm resolutions across all field strengths tested. Differences begin to appear at the 0.5 mm resolution, particularly at object boundaries, likely due to discrete object approximation affecting the off‐resonance calculations at implant edges (purple arrows). The impact of discretization becomes more pronounced at higher field strengths, where off‐resonance effects are proportionally larger (blue arrow). The 0.2 mm phantom provides better differentiation between ripple artifacts and discretization artifacts, especially at 3 T (orange arrows). However, the 0.5 mm phantom is generally sufficient for the simulation of signal void artifacts at all field strengths. The 1 mm resolution shows substantial discretization artifacts compared to other resolutions (red arrows) and is generally insufficient for accurate simulation outcomes, though it may be used for quick preliminary assessments. Simulations with 0.1 mm phantoms require substantial computing resources, demanding approximately 4 days of computation time and 0.5 TB of memory per SEMAC 18 simulation, which may limit practical use. These conclusions are specific to this implant geometry and the chosen simulation setup, and similar analyses should be performed for different configurations.
**Figure S4:** Analysis of signal contributions from spectral bins. Bar plots show the percentage of signal within 3 cm of the implant outline (*y*‐axis) for each spectral bin (*x*‐axis) for the implant configurations of CoCr (top) and Titanium (bottom). Signal percentages are shown for 0.55 T (orange), 1.5 T (red), and 3 T (blue). The image on the right shows the region of interest (within 3 cm of the implant boundary) from which these signal percentages are calculated. At 0.55 T, signal contributions are concentrated in fewer spectral bins for both implant configurations, while at higher field strengths, signal is more distributed across the spectral range. The Titanium configuration shows more concentrated distribution compared to CoCr due to lower magnetic susceptibility.
**Figure S5:** Spectral bin images for CoCr implant configuration. Individual spectral bin images from a central slice for 36 spectral encodings at 0.55 T (top), 1.5 T (middle), and 3 T (bottom). The spectral bin frequency is shown in white text above each image, and the signal percentage within 3 cm of the implant boundary is shown in yellow text. These signal percentages correspond to the values plotted in the top bar plot of Figure S4. The images illustrate how signal recovery varies across different frequency bins and field strengths, with higher field strengths requiring more spectral bins to recover signal from off‐resonance spins around the CoCr implant. Signals spatially closer to the implant are recovered in the furthest spectral bins from the central frequency, although these bins contribute lower overall signal percentages.
**Figure S6:** Spectral bin images for Titanium implant configuration. Individual spectral bin images from a central slice for 36 spectral encodings at 0.55 T (top), 1.5 T (middle), and 3 T (bottom) for the Titanium/ceramic implant setup. The spectral bin frequency is shown in white text above each image, and the signal percentage within 3 cm of the implant boundary is shown in yellow text. These signal percentages correspond to the values plotted in the bottom bar plot of Figure S4. Compared to the CoCr configuration shown in Figure S5, the Titanium setup demonstrates more concentrated signal distribution due to reduced magnetic susceptibility effects. Signals spatially closer to the implant are recovered in the furthest spectral bins from the central frequency, although these bins contribute lower overall signal percentages.

## Data Availability

Source code is provided at https://github.com/usc‐mrel/mri_metal_simulation. Phantom data are provided at https://doi.org/10.5281/zenodo.16003438.
