# Supplementary material for: Open‐Source Simulator of Imaging Near Metal at Arbitrary Magnetic Field Strengths
Source: Magn Reson Med. 2025 Nov 3;95(4):2370–83. doi: 10.1002/mrm.70163 (PMC12850566; doi:10.1002/mrm.70163)
Supplement: Supplementary file 1 — Figure S1: Quantitative evaluation of the artifact. (Left) Simulation with metal susceptibility showing the signal void artifact around the implant. (Middle) Simulation with identical parameters but implant susceptibility set to match human tissue, showing the expected signal without metal‐induced artifacts. (Right) Absolute difference image obtained by subtracting the tissue‐matched simulation from the metal simulation, emphasizing the artifact region appearing with intensities corresponding to the underlying tissue values. The positive signal visible at the location of the metal implant (indicated by purple arrow) is due to undersampling in the spectral domain, which causes aliasing artifacts along the spectral dimension. Artifact right–left extent and superior–inferior extent measure the maximum horizontal and vertical dimensions of the signal void, while artifact area quantifies the total two‐dimensional area affected by the signal loss. The measurement regions for area (red overlay) and length extents (yellow and blue dashed lines) are overlaid on the difference image. Artifact measurements were performed by manual tracing of visually apparent signal void boundaries in the difference images. These measurements are compared against the actual implant dimensions to determine the degree of artifact sizes at different field strengths and sequence parameters. Figure S2: Illustration of the effect of implant material on artifact size at 0.55, 1.5, and 3 T. Comparison of metal artifacts between two different implant head configurations: one with CoCr components using SEMAC 12 and another with Titanium/ceramic components using SEMAC 6. Despite the Titanium setup using fewer spectral encodings, it consistently produces smaller artifacts than the CoCr implant at all field strengths due to its lower magnetic susceptibility, and the artifacts are predominantly localized around the neck of the implant (orange arrows). The difference between materials becomes more pronounced [file MRM-95-2370-s001.docx]

# Supporting Information

**
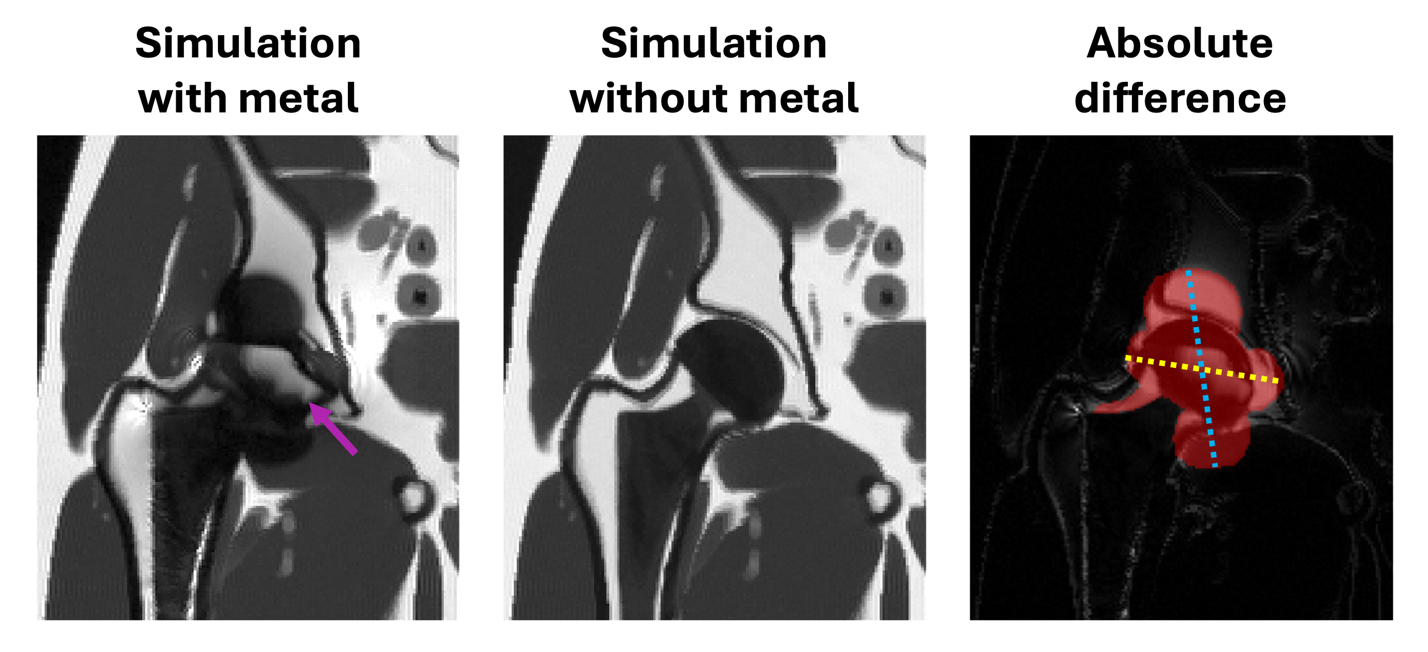
**

**Figure S1: Quantitative evaluation of the artifact.** (Left) Simulation with metal susceptibility showing the signal void artifact around the implant. (Middle) Simulation with identical parameters but implant susceptibility set to match human tissue, showing the expected signal without metal-induced artifacts. (Right) Absolute difference image obtained by subtracting the tissue-matched simulation from the metal simulation, emphasizing the artifact region appearing with intensities corresponding to the underlying tissue values. The positive signal visible at the location of the metal implant (indicated by purple arrow) is due to undersampling in the spectral domain, which causes aliasing artifacts along the spectral dimension. Artifact right-left extent and superior-inferior extent measure the maximum horizontal and vertical dimensions of the signal void, while artifact area quantifies the total two-dimensional area affected by the signal loss. The measurement regions for area (red overlay) and length extents (yellow and blue dashed lines) are overlaid on the difference image. Artifact measurements were performed by manual tracing of visually apparent signal void boundaries in the difference images. These measurements are compared against the actual implant dimensions to determine the degree of artifact sizes at different field strengths and sequence parameters.


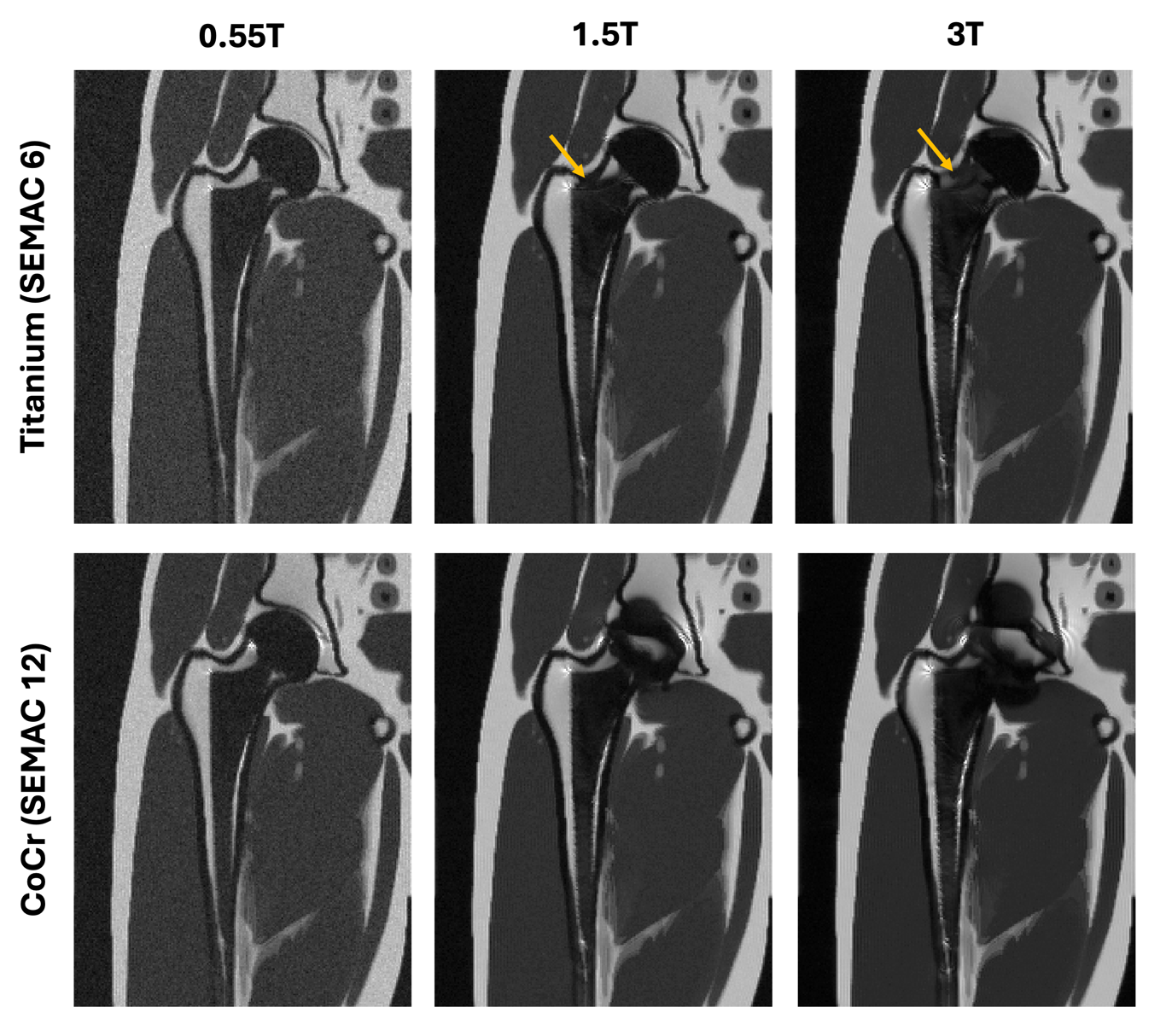


**Figure S2:** **Illustration of the effect of implant material on artifact size at 0.55T, 1.5T, and 3T.** Comparison of metal artifacts between two different implant head configurations: one with CoCr components using SEMAC 12 and another with Titanium/ceramic components using SEMAC 6. Despite the Titanium setup using fewer spectral encodings, it consistently produces smaller artifacts than the CoCr implant at all field strengths due to its lower magnetic susceptibility, and the artifacts are predominantly localized around the neck of the implant (orange arrows). The difference between materials becomes more pronounced at higher fields, with 3T showing the largest disparity in artifact size. This demonstrates that lower susceptibility materials allow for fewer spectral encodings and, therefore, shorter scan times.

**
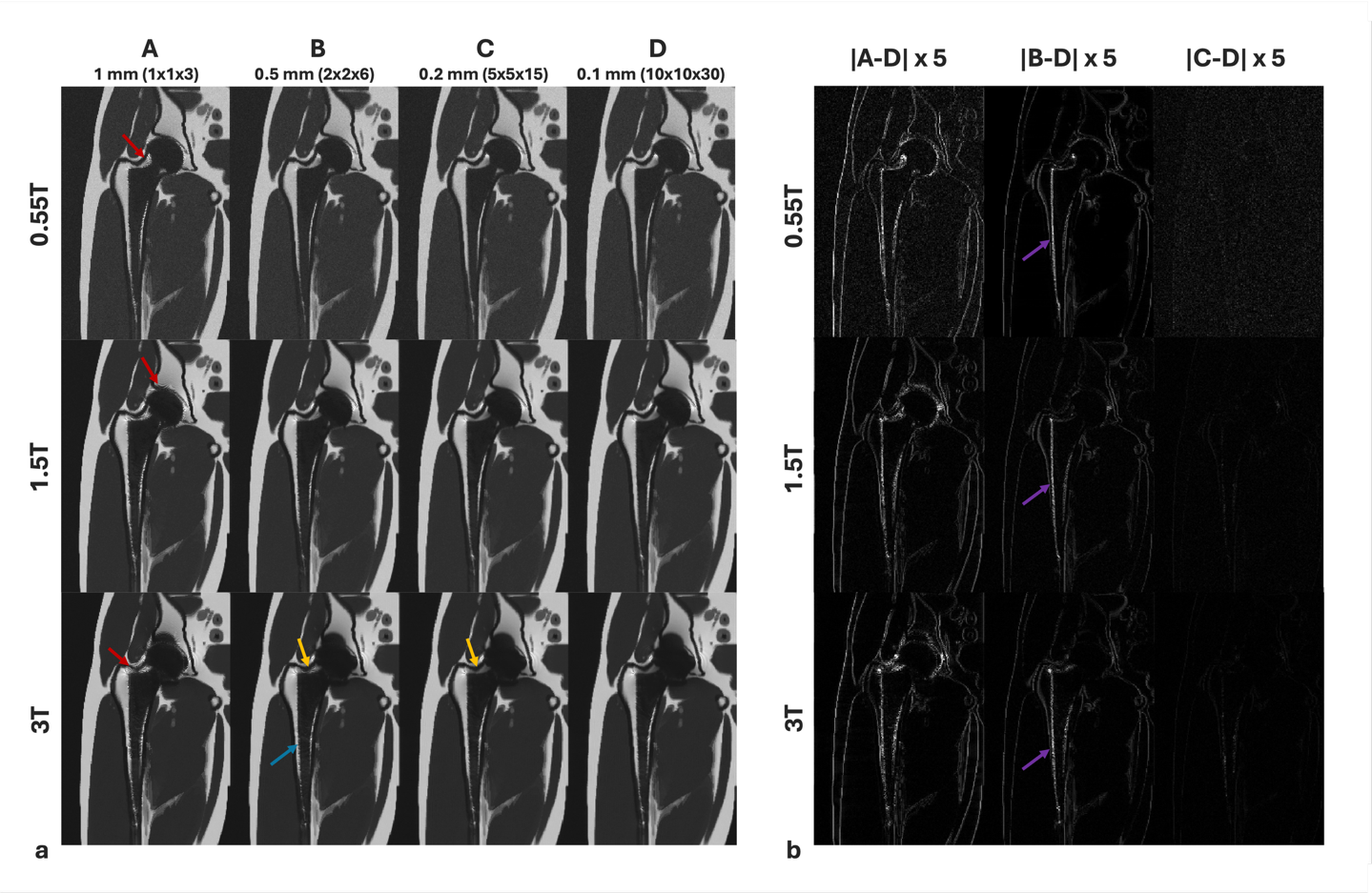
**

**Figure S3:** **Impact of phantom resolution on simulation accuracy.** **(a)** Simulation of SEMAC images with 18 spectral encodings using phantom resolutions of 1 mm, 0.5 mm, 0.2 mm, and 0.1 mm isotropic (1x1x3, 2x2x6, 5x5x15, and 10x10x30 times finer than imaging resolution, respectively) at field strengths of 0.55T, 1.5T, and 3T. **(b)** Absolute differences between the finest resolution (0.1 mm) and other resolutions (1 mm, 0.5 mm, and 0.2 mm) (shown with 5x enhancement) to identify regions where phantom resolution significantly impacts simulation results at each field strength. No significant differences are observed between images simulated with 0.1 mm and 0.2 mm resolutions across all field strengths tested. Differences begin to appear at the 0.5 mm resolution, particularly at object boundaries, likely due to discrete object approximation affecting the off-resonance calculations at implant edges (purple arrows). The impact of discretization becomes more pronounced at higher field strengths, where off-resonance effects are proportionally larger (blue arrow). The 0.2 mm phantom provides better differentiation between ripple artifacts and discretization artifacts, especially at 3T (orange arrows). However, the 0.5 mm phantom is generally sufficient for the simulation of signal void artifacts at all field strengths. The 1 mm resolution shows substantial discretization artifacts compared to other resolutions (red arrows) and is generally insufficient for accurate simulation outcomes, though it may be used for quick preliminary assessments. Simulations with 0.1 mm phantoms require substantial computing resources, demanding approximately 4 days of computation time and 0.5 TB of memory per SEMAC 18 simulation, which may limit practical use. These conclusions are specific to this implant geometry and the chosen simulation setup, and similar analyses should be performed for different configurations.

**
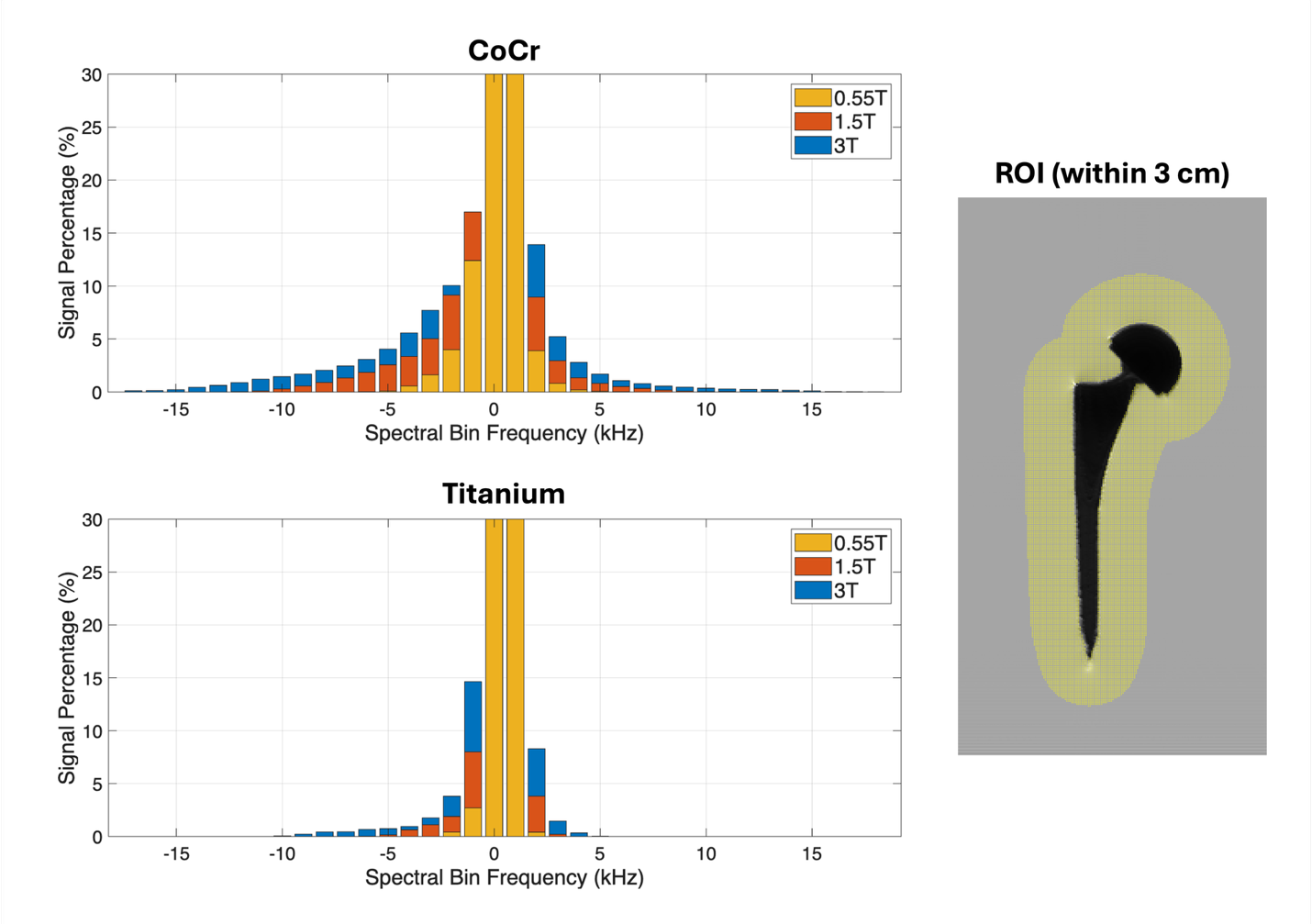
**

**Figure S4:** **Analysis of signal contributions from spectral bins. Bar plots show the percentage of signal within 3 cm of the implant outline (y-axis) for each spectral bin (x-axis) for the implant configurations of CoCr (top) and Titanium (bottom). Signal percentages are shown for 0.55T (orange), 1.5T (red), and 3T (blue). The image on the right shows the region of interest (within 3 cm of the implant boundary) from which these signal percentages are calculated. At 0.55T, signal contributions are concentrated in fewer spectral bins for both implant configurations, while at higher field strengths, signal is more distributed across the spectral range. The Titanium configuration shows more concentrated distribution compared to CoCr due to lower magnetic susceptibility.**


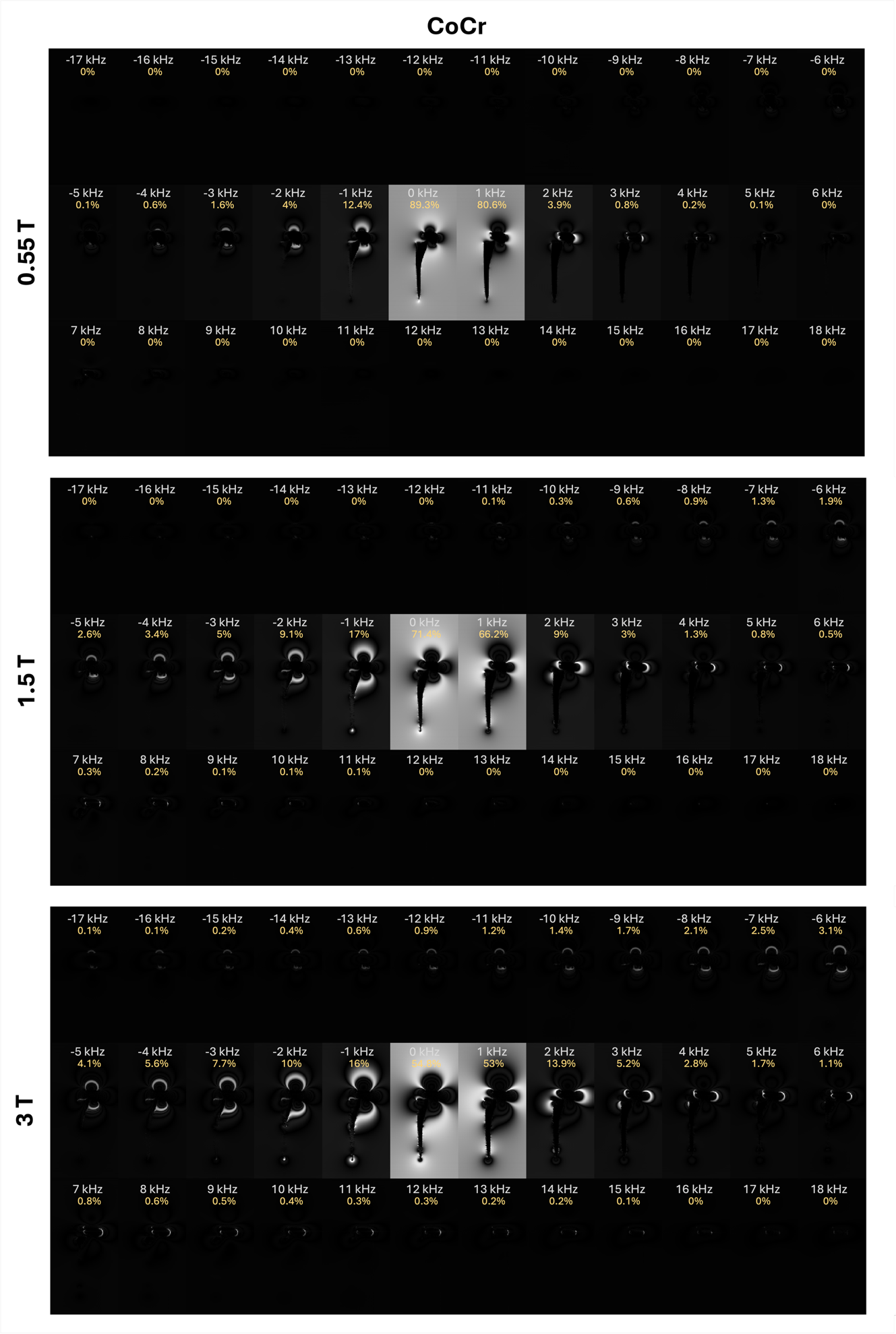


**Figure S5:** **Spectral bin images for CoCr implant configuration.** Individual spectral bin images from a central slice for 36 spectral encodings at 0.55T (top), 1.5T (middle), and 3T (bottom). The spectral bin frequency is shown in white text above each image, and the signal percentage within 3 cm of the implant boundary is shown in yellow text. These signal percentages correspond to the values plotted in the top bar plot of **Figure S4**. The images illustrate how signal recovery varies across different frequency bins and field strengths, with higher field strengths requiring more spectral bins to recover signal from off-resonance spins around the CoCr implant. Signals spatially closer to the implant are recovered in the furthest spectral bins from the central frequency, although these bins contribute lower overall signal percentages.


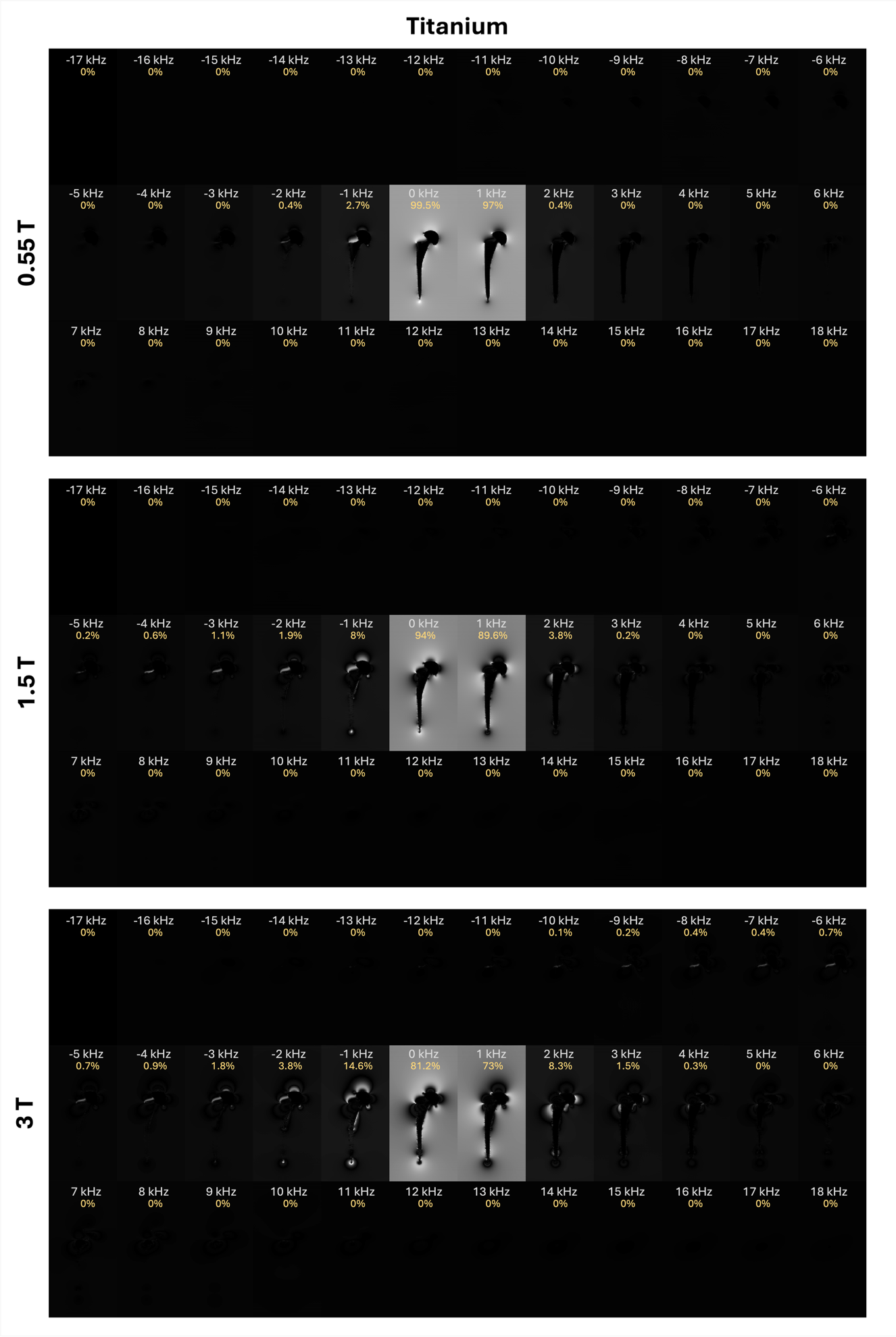


**Figure S6:** **Spectral bin images for Titanium implant configuration.** Individual spectral bin images from a central slice for 36 spectral encodings at 0.55T (top), 1.5T (middle), and 3T (bottom) for the Titanium/ceramic implant setup. The spectral bin frequency is shown in white text above each image, and the signal percentage within 3 cm of the implant boundary is shown in yellow text. These signal percentages correspond to the values plotted in the bottom bar plot of **Figure S4**. Compared to the CoCr configuration shown in **Figure S5**, the Titanium setup demonstrates more concentrated signal distribution due to reduced magnetic susceptibility effects. Signals spatially closer to the implant are recovered in the furthest spectral bins from the central frequency, although these bins contribute lower overall signal percentages.
